# Multi-Mode Coupling Enabled Broadband Coverage for Terahertz Biosensing Applications

**DOI:** 10.3390/bios15060368

**Published:** 2025-06-07

**Authors:** Dongyu Hu, Mengya Pan, Yanpeng Shi, Yifei Zhang

**Affiliations:** 1 School of Integrated Circuits, Shandong University, Jinan 250100, China; 202200400050@mail.sdu.edu.cn (D.H.); 202432401@mail.sdu.edu.cn (M.P.); yifeizhang@sdu.edu.cn (Y.Z.); 2 Shandong Key Laboratory of Metamaterial and Electromagnetic Manipulation Technology, Jinan 250100, China

**Keywords:** BIC and QBIC, multipolar hybridized modes, small angular variations, high quality factor, broadband terahertz sensing

## Abstract

Terahertz (THz) biosensing faces critical challenges in balancing high sensitivity and broadband spectral coverage, particularly under miniaturized device constraints. Conventional quasi-bound states in the continuum (QBIC) metasurfaces achieve high quality factor (Q) but suffer from narrow bandwidth, while angle-scanning strategies for broadband detection require complex large-angle illumination. Here, we propose a symmetry-engineered, all-dielectric metasurface that leverages multipolar interference coupling to overcome this limitation. By introducing angular perturbation, the metasurface transforms the original magnetic dipole (MD)-dominated QBIC resonance into hybridized, multipolar modes. It arises from the interference coupling between MD, toroidal dipole (TD), and magnetic quadrupole (MQ). This mechanism induces dual counter-directional, frequency-shifted, resonance branches within angular variations below 16°, achieving simultaneous 0.42 THz broadband coverage and high Q of 499. Furthermore, a derived analytical model based on Maxwell equations and mode coupling theory rigorously validates the linear relationship between frequency splitting interval and incident angle with the Relative Root Mean Square Error (RRMSE) of 1.4% and the coefficient of determination ($R^{2}$) of 0.99. This work establishes a paradigm for miniaturized THz biosensors, advancing applications in practical molecular diagnostics and multi-analyte screening.

## 1. Introduction

Terahertz (THz) band refers to electromagnetic waves ranging from 0.1 to 10 THz bands between microwave and infrared bands [1]. Due to its label-free and associated unique properties, THz technology has emerged as a promising platform for biosensing detection [2,3,4,5]. Nevertheless, the mismatch between THz wavelengths and the size of biomedical analytes results in weak light–matter interactions, leading to limited sensitivity [6,7]. In order to address this issue, metallic metasurfaces with localized field enhancement have been employed to amplify THz–sample interactions [8]. For example, Zhang et al. [9] proposed a metasurface structure composed of metal split ring resonators and obtained three modes of localized field enhancement. Gao et al. [10] reported a two-layer metasurface comprising copper bars, Dirac semimetal materials, and a silicon substrate, achieving a quality factor (Q) of 147. Qi et al. [11] proposed a metasurface composed of two periodically arranged joint split rings for sensing applications, and its sensitivity reached 200 GHz/RIU. Although these methods enhance the localized field, conventional metallic architectures suffer from inherent Ohmic losses that limit further improvement in Q and sensing performance [12]. In contrast, dielectric metamaterials have emerged as a low-loss alternative. Yan et al. [13] reported a C_4v_-symmetric dielectric THz metasurface, exciting a magnetic dipole (MD) mode with an ultrahigh sensitivity of 446 GHz/RIU. Despite reduced material losses, radiation losses in dielectric systems continue to limit performance metrics. This necessitates novel physical mechanisms for next-generation THz sensing.

Bound states in the continuum (BIC), characterized by theoretically infinite Q and vanishing resonance linewidths, behaves as a special mode and offers a promising solution [14,15,16,17]. As THz waves are focused by the metasurface into an extremely confined volume, BIC indicates great enhancement of light–matter interactions, which show high sensitivity to tiny environmental changes [18,19,20]. Ideal BIC remains decoupled from free space and is thus impractical for sensing [21,22]. In practical applications, an ideal BIC mode is transformed into a quasi-BIC (QBIC) mode with an ultrahigh Q and finite resonance linewidths because of the material losses and other perturbations in the metasurfaces system [23,24,25]. Dong et al. demonstrated a simulated refractive index sensitivity of up to 544 GHz/RIU by exciting QBIC through a dual-chain-separated, asymmetric resonant cavity structure [26]. Liu et al. manipulated the interference coupling between the electric quadrupole and MD by introducing an asymmetry into the metasurface, which excites QBIC resonance with a Q of up to 503 [27]. Nevertheless, conventional QBIC sensors face spectral incompatibility challenges. The narrow linewidth of resonance makes it difficult to cover the analyte fingerprint spectrum [28]. Zhong et al. utilized an angle-scanning strategy to broaden the reflection spectrum bandwidth of their metasurface sensor to 0.832–1.05 THz, covering the absorption peak of tyrosine and santonin [29]. By increasing the incident angle from 0° to 40°, the metasurface proposed by Sun et al. achieves a broadband spectral coverage of 1.74–2.14 THz, enabling the simultaneous identification of melamine and vanillin [30]. However, such large-angle illumination complicates optical alignment and prolongs measurement duration. The development of miniaturized THz biosensors capable of broadband detection under small angular variations remains an open challenge in the field.

In this paper, we propose a symmetry-engineered, all-dielectric metasurface to simultaneously achieve high Q resonance and broadband under small angular variations. The unit cell integrates dual elliptical silicon resonators with chevron-like geometry on a calcium fluoride (CaF_2_) substrate, where symmetry breaking enables the transformation of a MD-dominated QBIC into a hybridized state. Specifically, the introduction of angular perturbations induces interference coupling between toroidal dipole modes (TD) and magnetic quadrupoles modes (MQ). It dynamically splits the original single resonance peak into dual counter-directional spectral branches: a red-shifted resonance dominated by the MD mode and a blue-shifted resonance by hybridized multipolar excitations. This multipolar interference coupling mechanism achieves a broadband coverage of 0.42 THz and a high Q of 499 under a small-angle scanning strategy from 0° to 16°. In addition, a derived analytical model is introduced. It is grounded in Maxwell equations and angle-dependent mode coupling theory, rigorously validating the linear relationship between the frequency splitting interval and the incident angle. Our approach eliminates the need for large-angle illumination, thereby simplifying optical alignment and reducing measurement complexity, paving the way toward the development of miniaturized THz biosensors capable of broadband detection.

## 2. Materials and Methods

**Figure 1:**
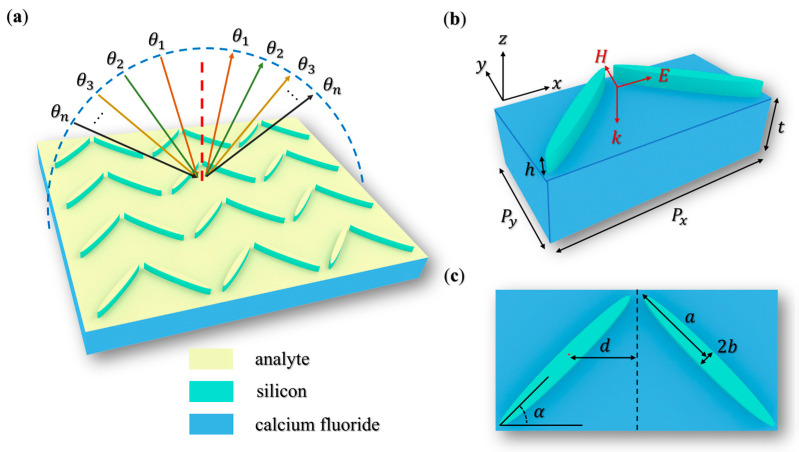
(**a**) Schematic diagram of the QBIC metasurface platform, illuminated by THz waves at variable incidence angles *θ*. (**b**) Unit cell architecture with periodic boundary conditions and wave excitation parameters. Arrows indicate z-propagating wavevector and x-polarized electric field. (**c**) Dimensional schematic of the dual elliptical cylinder resonators.

Figure 1a shows the BIC metasurface platform composed of silicon dual elliptical cylinder resonators on a CaF_2_ substrate. The beige, thin-layer structure is the analyte covering the metasurface. The unit cell configuration of the metasurface is labeled in Figure 1b with lattice constants *P_x_* = 288 µm, *P_y_* = 144 µm, and thickness *t* = 80 µm. The system is illuminated by a linearly polarized plane wave in the 0.6–1.4 THz range, propagating along the *z*-axis with an x-polarized electric field. Figure 1c details the geometric parameters of the elliptical resonator. The silicon resonators exhibit ±45° rotational alignment relative to the lattice axes, creating a chevron-like symmetry along the y–z plane. Each element has a semi-major axis *a* = 90 µm, semi-minor axis *b* = 9 µm, and height *h* = 20 µm. The resonator pair is displaced by *d* = 72 µm from the unit cell origin, forming a symmetry-modified configuration. This geometric design can be achieved through lithographic patterning and anisotropic etching [31], with correlated angular positional tolerance margins designed to enhance system robustness. Specifically, angular adjustments within this range dynamically compensate for positional deviations through geometric phase modulation, ensuring stable, near-field interactions under fabrication variations. The elliptical aspect ratio (*a*/*b* = 10) and lattice periodicity ratio (*P_x_*/*P_y_* = 2) collectively establish the anisotropic optical response of the system.

The electromagnetic response of the metasurface is numerically investigated using three-dimensional, finite-difference time-domain (3D FDTD) simulations. The computational boundaries are configured with perfectly matched layers along the z-direction to suppress numerical reflections, while periodic boundary conditions incorporating phase correction algorithms are applied in both x- and y-directions to maintain lattice periodicity. This configuration rigorously replicates the infinite-array behavior of the metasurface under plane wave excitation. The material dispersion of silicon resonators is modeled using a constant refractive index approximation (n = 3.42) across the operational wavelength range, while the CaF_2_ substrate (n = 1.38) is treated as a lossless dielectric medium. A non-uniform meshing scheme with subwavelength resolution ensures convergence of near-field enhancement and far-field radiation patterns.

## 3. Results and Discussion

**Figure 2:**
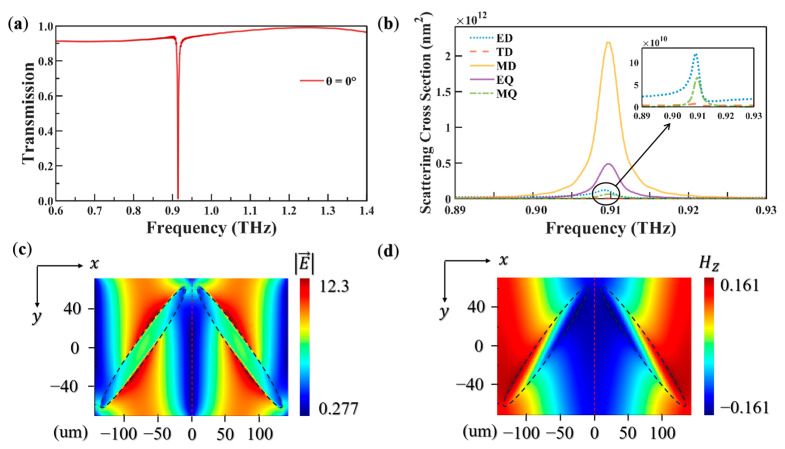
(**a**) Transmission spectrum of the metasurface under vertical incidence. (**b**) Scattering spectra of dual-silicon elliptical cylinder resonators under vertical incidence (ED is electrical dipole and EQ is electric quadrupole). (**c**) Electric field distribution in the x–y plane at the resonance frequency. (**d**) Magnetic field distribution in the x–y plane at the resonance frequency.

Figure 2a illustrates the transmission spectrum of the metasurface under vertical incidence. It exhibits a prominent transmission peak at 0.914 THz and a high Q of 499. The scattering cross-sections were rigorously calculated through Cartesian multipole decomposition, where higher-order terms beyond the dipole approximation were neglected [32,33]. As evidenced by the multipolar expansion in Figure 2b, the MD resonance dominates the scattering response at this frequency, accounting for 77.59% of the total cross-section. Figure 2c,d depict the corresponding near-field electric and magnetic distributions in the x–y plane. Under x-polarized electric field excitation, a toroidal current distribution emerges within the elliptical cylinder resonators. Owing to its structural symmetry, the induced circulating currents in adjacent ellipses exhibit identical rotational orientations. This configuration drives antiparallel equivalent surface currents along the neighboring walls, generating mutual repulsion effects. Therefore, the electric field is suppressed at the endpoints of the major axis and primarily confined to the central region of the elliptical cylinder walls away from the major axis endpoints. This spatial redistribution is governed by charge–repulsion dynamics. The toroidal current within the elliptical cylinder generates a MD moment along the *z*-axis. The magnetic field distribution reveals that the vertical component *Hz* exhibits alternating polarity within the ellipse, forming a doughnut-shaped field topology. This behavior aligns with the characteristics of MD resonance [34]. Notably, higher-order multipoles remain suppressed due to geometric symmetry constraints.

**Figure 3:**
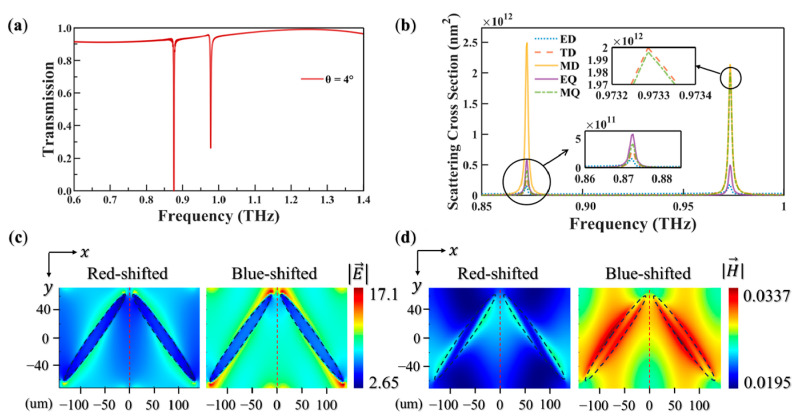
(**a**) Transmission spectrum of the metasurface under oblique incidence *θ* = 4°. (**b**) Scattering spectra of dual-silicon elliptical cylinder resonators under oblique incidence *θ* = 4°. (**c**) Electric field distributions in the x–y plane at both red-shifted and blue-shifted resonances. (**d**) Magnetic field distributions in the x–y plane at both red-shifted and blue-shifted resonances.

Figure 3a demonstrates that under oblique incidence *θ* = 4°, the originally single transmission peak undergoes splitting into dual counter-directional resonances. Specifically, the resonance exhibits red-shifted 0.875 THz and blue-shifted 0.977 THz spectral branches, with 0.914 THz serving as the reference frequency. This phenomenon originates from the symmetry-breaking effect of the metasurface. The mirror symmetry along the y–z plane restricts excitation to the low-order mode at vertical incidence, whereas oblique incidence perturbs this symmetry, enabling higher-order modes excitation. The periodicity of the metasurface requires momentum conservation governed by equation [35]:

(1) $$k_{inc,x}+m\cdot \frac{2\pi }{a_{x}}=k_{Bloch,x}$$

where $k_{inc,x}$ is the tangential component of the incident wave vector, $k_{Bloch,x}$ is the Bloch wave vector of the periodic structure, and $a_{x}$ is the period of the array in the x-direction.

For vertical incidence $k_{inc,x}$ = 0, this equation simplifies to $k_{Bloch,x}$ = $2\pi m/a_{x}$, allowing only the m = 0 Bloch mode, called even-symmetric field distribution. Higher-order modes (m ≠ 0) remain symmetry-forbidden. For oblique incidence $k_{inc,x}$ = ω/c·sin⁡θ, the equation is modified into

(2) $$k_{Bloch,x}=\frac{\omega }{c}\sin\theta +m\cdot \frac{2\pi }{a_{x}}$$

It permits excitation of multiple Bloch harmonics (m = 0, ±1), releasing the mode degeneracy and splitting the resonance. The red-shifted resonance corresponds to the m = 0 mode, while the blue-shifted resonance arises from *m* = ±1 harmonics with higher-order modes. Figure 3b depicts scattering cross-section characteristics. The red-shifted resonance at 0.875 THz maintains MD dominance, mirroring the vertical incidence configuration. Conversely, the blue-shifted resonance at 0.977 THz manifests as a cooperative interplay of three principal components with MD (31.43%), TD (29.40%), and MQ (29.36%), collectively constituting 89.19% of the total scattering response. Particularly noteworthy is the near-degenerate behavior between the TD and MQ components, exhibiting near-identical magnitude distributions. Figure 3c presents the electric field distributions at both red-shifted and blue-shifted resonances. At the red-shifted resonance, the electric field exhibits a dipole-like pattern akin to vertical incidence, with strong confinement along the elliptical cylinder walls. In contrast, the blue-shifted resonance displays a complex multipolar signature. The electric field intensity is maximized at the endpoints of the major axis of the elliptical cylinder, forming two antipodal hot spots. These results highlight the distinct spatial localization of the electric field at the two resonant modes. Figure 3d shows the corresponding magnetic field distributions. For the red-shifted resonance, the magnetic field forms a doughnut-shaped toroidal distribution encircling the structure, indicating a MD-dominated response. The blue-shifted resonance reveals a hybridized, higher-order multipolar mode. The magnetic field demonstrates dual flux-concentrated regions laterally aligned on both sides of the major axis, with strong localization along the elliptical cylinder walls. These magnetic flux zones exhibit tangential orientations parallel to the wall surface, consistent with the interplay of multipolar modes [36].

**Figure 4:**
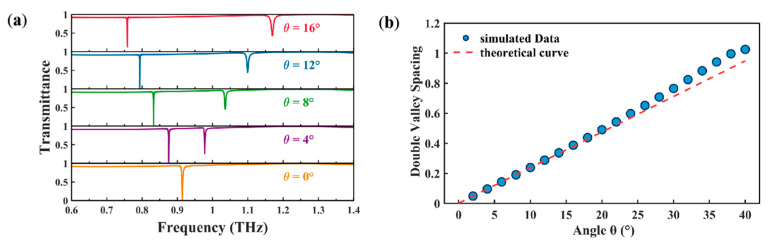
(**a**) Angle-dependent transmission spectra of metasurfaces across 0–16° incidence angles. (**b**) Comparison between simulated data and theoretical curve.

Figure 4a illustrates the impact of small angular scanning strategies on the metasurface transmission spectra. As the incident wavevector tilts from vertical to oblique incidence, the transmission characteristic shifts from a single peak to dual counter-directional resonance states. Progressive frequency splitting between these peaks establishes broadband spectral coverage. Quantitative analysis reveals that when *θ* increases from 0° to 16°, the total spectral span between split resonances reaches 0.42 THz, corresponding to a relative bandwidth of $\Delta f/f_{c}$ = 45.95% with $f_{c}$ = 0.914 THz as the reference frequency, where Δf denotes the frequency separation. This broadband behavior originates from the multi-mode coupling mechanism [37,38], as derived through Maxwell equations under periodic boundary conditions [39]:

(3) $$\omega_{\pm }=\frac{\left(\omega_{1}+\omega_{2}\right)-i(\gamma_{1}+\gamma_{2})}{2}\pm \frac{\sqrt{{\left[\left(\omega_{1}-\omega_{2}\right)-i(\gamma_{1}-\gamma_{2})\right]}^{2}+4{\left(\chi -i\sqrt{\gamma_{1}\gamma_{2}}\right)}^{2}}}{2}$$

where $\omega_{1}$ and $\omega_{2}$ are the dip frequencies of the two resonant modes, $\gamma_{1}$ and $\gamma_{2}$ are the decay rates of the two modes, χ represents the near-field coupling between the modes.

By incorporating geometric phase correction and using a Taylor expansion to simplify, we derive the modified frequency relation. The resulting frequency separation scales as

(4) $$∆f=\frac{\chi k_{x}}{\pi \sqrt{\mu \epsilon_{00}}}$$

where $k_{x}$ is the component of the incident wave vector in the x-direction, μ is the magnetic permeability, and $\epsilon_{00}$ is the equivalent permittivity of the metasurface.

Under small-angle approximation (θ≤16°), this model predicts a linear proportionality between Δf and *θ*, in excellent agreement with simulated data in Figure 4b with the Relative Root Mean Square Error (RRMSE) of 1.4%. The RRMSE is defined as [40]

(5) $$RRMSE=\frac{\sqrt{\frac{1}{n}\sum_{i=1}^{n}{\left(y_{i}-\hat{y_{i}}\right)}^{2}}}{\bar{y}}$$

where $y_{i}$ represents the simulated values, $\hat{y_{i}}$ denotes the predicted values, n is the number of data points, and $\bar{y}$ is the mean of the simulated values. Additionally, the coefficient of determination ($R^{2}$) further confirms a strong correlation, with an $R^{2}$ of 0.99, underscoring the model’s predictive power and reliability. The $R^{2}$ value is calculated as [41]

(6) $$R^{2}=1-\frac{\sum_{i=1}^{n}{\left(y_{i}-\hat{y_{i}}\right)}^{2}}{\sum_{i=1}^{n}{\left(y_{i}-\bar{y}\right)}^{2}}$$

When the incident angle is less than 16°, the absolute deviation between the simulation and theoretical values is less than 0.01, and the relative deviation is less than 2%. This confirms the accuracy of the linear approximation within this angular range [42]. However, as the incident angle increases beyond 16°, the deviations become more pronounced. At 38°, the absolute deviation reaches 0.1, and the relative deviation increases to 10%.

**Figure 5:**
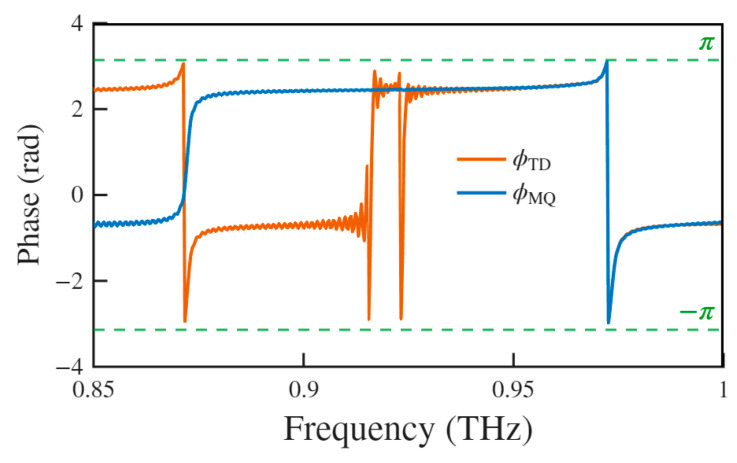
Analytical diagram of phase variations in TD and MQ resonances at *θ* = 4°. (ΦTD is phase variation in TD resonance and ΦMQ is phase variation in MQ resonance).

Figure 3a shows distinct transmittance differences between different modes. At the red-shifted resonance, the transmittance reaches T = 0.99, whereas at the blue-shifted resonance, it drops to T = 0.74. This disparity is governed by the frequency-dependent, multipole interference transmission mechanism. Figure 5 contains a detailed phase variations analysis, showing that the TD and MQ resonances maintain a locked π-phase difference near the red-shifted resonance. This stable phase difference induces complete destructive interference between their far-field radiation along the transmission axis, effectively eliminating scattering contributions from both multipoles. The consequent energy localization occurs through the non-radiative channel of the MD mode, directly explaining the observed transmission spectra. In the transitional frequency range preceding the blue-shifted resonance (0.9–0.95 THz), mode cross-coupling fundamentally alters the electromagnetic response of the metasurface. Near-field coupling disrupts the original resonance conditions, triggering phase oscillations in the TD mode. As the frequency approaches the blue-shifted resonance, these phase oscillations gradually cease. Eventually, phase synchronization is achieved between the TD and MQ modes, leading to the simultaneous excitation of TD and MQ modes and constructive interference in their radiation. Concurrently, the superposition of the original MD-derived field with hybridized multipolar components disrupts phase matching in the transmission channel. The resulting tripartite TD-MQ-MD hybrid resonance activates a dual dissipation mechanism, significantly enhancing far-field scattering losses. These coordinated effects produce the characteristic transmittance suppression at the blue-shifted resonance, with simulated quantification confirming a 25% reduction in transmission efficiency compared to the red-shifted resonance. The mechanism establishes a theoretical foundation for developing low-loss THz devices. Future advancements in structural symmetry engineering combined with targeted suppression of loss channels will enable precise control over multipolar phase relationships and mode competition, potentially leading to broadband and high Q metasurfaces.

The broadband, multi-resonance characteristics of our metasurface offer unique advantages for biosensing applications. First, the widened spectral range (0.75–1.17 THz) enables the simultaneous detection of multiple molecular ‘fingerprint’ absorption peaks, which is critical for distinguishing biomolecules with overlapping spectral features. Second, our design fundamentally addresses the angular complexity bottleneck in miniaturized biosensors. Conventional spectroscopic systems requiring large-angle illumination (>35°) [43] face severe challenges in integration due to their dependence on high-resolution stepper motors and multi-axis mechanical structures [44]. In contrast, our metasurface achieves 0.42 THz bandwidth coverage through angularly compact spectral tuning, requiring only 16° angle range. This reduction in angular scanning range significantly simplifies optical alignment. Third, the enhanced near-field resonance amplifies light–matter interactions, enabling more sensitive detection.

## 4. Conclusions

In summary, this study establishes a multi-mode, all-dielectric, metasurface platform for broadband THz fingerprint detection. Composed of symmetrical dual elliptical silicon resonators shaped like chevrons on the CaF_2_ substrate, the platform achieves simultaneous high Q resonance and 0.42 THz broadband coverage. Under vertical incidence, the metasurface exhibits a MD-dominated QBIC resonance at 0.914 THz with a Q of up to 499. Oblique incidence induces momentum–space reconstruction, splitting the single resonance into dual counter-directional resonance branches. By utilizing a small angle-scanning strategy from 0° to 16°, our metasurface achieves a wide frequency coverage from 0.75 THz to 1.17 THz. The key to this resonance splitting phenomenon lies in the multipolar interaction coupling under symmetry breaking. At the red-shifted resonance, the locked π-phase difference between the TD and MQ modes concentrates energy on the MD mode. At the blue-shifted resonance, hybridized TD-MQ-MD interactions activate dual dissipation channels. These phenomena are unified under an angular-frequency-dependent, multipolar interference framework, where structural symmetry dictates mode competition and energy partitioning between radiative/non-radiative pathways. Furthermore, a derived analytical model is introduced. It is grounded in Maxwell equations and angle-dependent mode coupling theory, rigorously validating the linear relationship between the frequency splitting interval and the incident angle. The demonstrated principles—symmetry-mediated QBIC formation, Bloch wave-engineered broadband expansion, and angular multipolar interference coupling—establish a transformative framework for next-generation biosensing systems. By synergistically combining angularly compact spectral tuning—achieving 0.42 THz bandwidth coverage within a 16° angular range—with geometrically enhanced near-field localization, our platform enables two fundamental breakthroughs in biomolecular detection. First, the simultaneous resolution of multiple molecular fingerprint absorption peaks permits multiparametric analyte characterization through single spectroscopic measurements, effectively addressing spectral overlap challenges in complex biological matrices. Second, the elimination of bulky, large, angular scanning mechanisms facilitates direct integration with miniaturized THz biosensors systems, fulfilling critical requirements for portable point-of-care diagnostic devices. These dual capabilities collectively position our technology as a paradigm-shifting solution for label-free, high-throughput biomolecular analysis across clinical and environmental monitoring applications.

## Data Availability

The data underlying the results presented in this paper may be obtained from the authors upon reasonable request.
